# Autochthonous Hepatitis E during Pregnancy, France

**DOI:** 10.3201/eid2408.180105

**Published:** 2018-08

**Authors:** Elise Bouthry, Alexandra Benachi, Alexandre J. Vivanti, Emmanuelle Letamendia, Christelle Vauloup-Fellous, Anne-Marie Roque-Afonso

**Affiliations:** Assistance Publique Hôpitaux de Paris, Hôpital Paul Brousse, Villejuif, France (E. Bouthry, C. Vauloup-Fellous, A.-M. Roque-Afonso);; Groupe de Recherche sur les Infections pendant la Grossesse, Clamart, France (E. Bouthry, C. Vauloup-Fellous, A.-M. Roque-Afonso);; Assistance Publique Hôpitaux de Paris, Hôpital Antoine Béclère, Clamart (A. Benachi, A.J. Vivanti, E. Letamendia);; Université Paris-Sud, Villejuif (C. Vauloup-Fellous, A.-M. Roque-Afonso)

**Keywords:** hepatitis E, HEV, pregnancy, mother-to-child transmission, genotype 3, viruses, France

## Abstract

Acute hepatitis E virus infections occurred during the third trimester in 2 pregnant women in France who sought treatment with nonspecific symptoms or asymptomatic elevation of liver enzymes. Infection cleared quickly in both women. We detected no hepatitis E RNA in 1 newborn’s feces at 3 weeks of age.

In low-income countries, estimates of 20 million hepatitis E virus (HEV) infections and 70,000 deaths have been reported for 2005. Increased mortality rate in pregnant women is well described but unexplained; pregnant women with symptomatic HEV infection have a 10-fold higher probability of death, especially in countries of the Indian subcontinent and Africa in which genotypes 1 and 2 are prevalent (*1*). In addition, up to 50% mother‐to‐child transmission rates have been reported, resulting in both premature births and prenatal deaths (*2*). In high-income countries with high seroprevalence rates, infection is usually asymptomatic. Most symptomatic cases are caused by HEV genotype 3 (*3*); illness ranges from mild to fulminant acute hepatitis, as well as chronic hepatitis in immunocompromised patients. No increased severity has been observed in the few cases reported during pregnancy (*4*–*8*).

We report 2 infections acquired during pregnancy in immunocompetent women in France, neither of whom reported having traveled abroad or eaten raw or undercooked pork. In accordance with hospital policy, patients were informed at hospital admission of the potential use of their anonymized medical data for research purposes. 

The first case-patient was a 43-year-old woman, hospitalized at 39 weeks 2 days’ gestation, who had nausea, headache, and increased serum alanine aminotransferase (ALT) (Figure). Hepatitis led to a cervical ripening and labor induction on the next day. At 39 weeks 5 days’ gestation, she delivered a 3,040-g healthy baby. We diagnosed maternal HEV infection at admission by detectable IgM and HEV RNA (5.75 log IU/mL), with a 3c genotype. Maternal cytolysis decreased significantly after 2 days in the hospital (from 600 to 300 U/L); physical examination remained normal in the weeks after delivery, so no further biologic follow-up was performed. At birth, the baby had normal liver enzymes; HEV RNA in stools was negative twice at birth and at weeks 2 and 3. 

**Figure:**
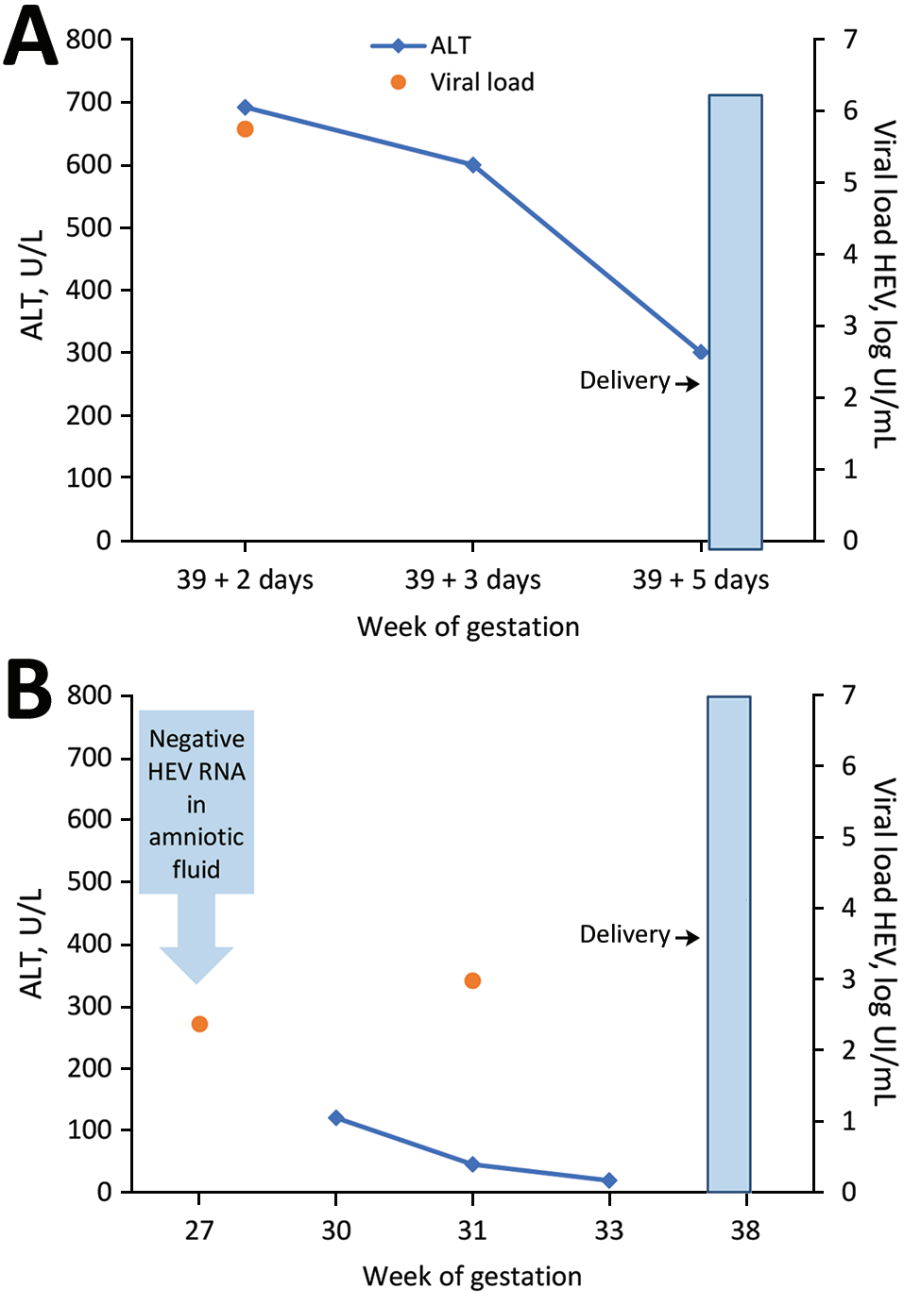
Relationship of HEV RNA and alanine aminotransferase to gestation and delivery time for 2 pregnant women, case-patient 1 (A) and case-patient 2 (B), France. Upper limit of normal for ALT values is 40 U/L. HEV RNA was quantified by a commercial real-time reverse transcription PCR assay targeting open reading frame 2/3. ALT, alanine aminotransferase; HEV, hepatitis E virus (Ceeram, La Chapelle sur Erdre, France).

The second case-patient was a 38-year-old woman with intrauterine growth restriction found at 24 weeks’ gestation. Increased ALT at 30 weeks’ gestation led to HEV diagnosis with detectable HEV IgM and viremia (2.98 log IU/mL). In retrospective testing, we detected HEV RNA in blood (2.37 log IU/mL) but not in the amniotic fluid at the time of amniocentesis (27 weeks’ gestation). HEV genotyping was unsuccessful. The patient’s liver abnormalities resolved by 33 weeks’ gestation. We performed no fetal or neonatal monitoring. The woman delivered a low-weight (2,350-g) but otherwise healthy baby at 38 weeks’ gestation. The maternal physical examination remained normal in the weeks after delivery; we performed no further hepatitis follow-up. 

Both patients had nonspecific symptoms or asymptomatic ALT elevation despite acute HEV infections in the third trimester of pregnancy. The rapid decline in ALT suggested a rapid clearance of infection in both. The lack of HEV RNA in the feces of 1 newborn indicated that infection was not transmitted; the lack of detectable RNA in the amniotic fluid suggested the same in the second case. We cannot rule out fetal HEV infection in patient 2 after 27 weeks’ gestation, even though such an infection is unlikely because both children were born healthy. These mild courses of illness are similar to previous reported outcomes involving HEV genotype 3 (*4*,*5*,*7*,*8*) but dissimilar to the findings of a retrospective case series from Israel (*6*). That study reported significant illness in 8 autochthonous cases: indeed, all but 1 case required hospitalization (from 1 week to 2 months in duration), and fulminant hepatitis was described in 2 postpartum cases. HEV genotyping was not available in this study. Poor outcomes, including spontaneous abortions, stillbirths, and premature delivery, have been related to impaired maternal immune and hormonal responses but have been limited to genotype 1. Genotype 1 replication has been shown in human placenta (*9*), which suggests a major role of the viral genotype in pathogenesis. This replication may account for the high rates of mother‐to‐child transmission and poor fetal outcomes in highly HEV-endemic areas. Recently, HEV infection in a rabbit model also demonstrated vertical transmission (*10*). However, rabbit HEV is a distant member of genotype 3 that causes only partial cross-species infections in nonhuman primates; thus, pathogenesis could be different for this specific HEV genotype 3 variant. We observed no mother-to-child transmission in the 2 cases we describe.

HEV infection among pregnant women in industrialized countries is rarely reported, perhaps because it is rare in this setting, or infection may be underdiagnosed because symptoms are mild or nonspecific. Indeed, no acute infection or seroconversions were observed among pregnant women in a prospective study in France (*11*). The cases we report further suggest that no maternal, fetal, or neonatal complications occur following HEV genotype 3 infection.
